# Pivot Teeth

**Published:** 1873-11

**Authors:** E. D. Swain


THE
### AMERICAN JOURNAL
OF
### DENTAL SCIENCE.

Vol. VII. THIRD SERIES--NOVEMBER, 1873. No. 7.

### ARTICLE I.

### *Pivot Teeth.*

BY DR. E. D. SWAIN.

The painless extraction of teeth, together with the easy and cheap methods now in vogue of inserting artificial denture upon plates, have the tendency largely to throw the attachment of artificial crowns to natural roots, by means of pivots, into disuse; though, under favorable circumstances, this is admitted to be one of the best modes that can be adopted. If the roots on which they are placed be sound and healthy, or if not healthy, made so, and the back teeth be reasonably good—that too much strain may not be thrown upon the pivoted tooth—these will subserve the purpose of the natural organs more perfectly than any other description of dental substitutes, and can be made to present an appearance so natural as to escape detection upon the closest scrutiny, as well as to render valuable service for many years. In short, their advantages may be summed up as follows: A better preservation of the contour of the parts surrounding; nearer approximation to nature; greater firmness, and therefore more serviceable, and greater economy for the wearer; besides giving to the operator, when successful, a sense of having done that which is best for his patient as well as himself.

Prof. Austen, in the chapter upon this subject in Harris' "Principles and Practice," informs us that only the six anterior superior teeth should ever be pivoted, and questions the propriety of pivoting to the roots of lateral incisors unless of very good size; further he tells us that in no case should a lower incisor be placed upon a pivot, because of the great danger of ulceration, and their small size. Both these objections I consider to be without a good and reasonable foundation. Fortunately we are very seldom called upon to replace single teeth of the latter class; and my experience has been that ulceration, when in that condition, is as easily managed as in other single straight rooted teeth. If called upon, however, to insert an inferior incisor where the root was intact, I should consider the pivot superior in every sense to any other known method. Bicuspids (especially the superior) may be, and often are, successfully set in this manner.

One of the strongest arguments in favor of pivot teeth is the *retention* of the root, and thereby preventing absorption of the alveoli about the necks of adjoining teeth; the removing of one tooth or root is taking the keystone from the arch.

The result, we all know, in time, is the gradual elongation of the tooth, or apsorbtion of the socket of adjoining teeth, exposing the root and weakening their support, and even sometimes, when an artificial tooth has been constantly worn on a plate, the adjoining teeth incline toward each other, and thus disfigure an otherwise comely set of teeth.

In the days past, when the nutrition of the teeth was not so well understood as at present, when it was believed that a pulpless tooth was dead, and would, like any other foreign substance, be thrown out, men were perhaps excusable for extracting such teeth under all circumstances; but with our better knowledge, our duty is to preserve, and thereby keep together, the different parts of the human machine as long as possible. That a so-called devitalized tooth is not entirely dead, we all know; that they do die, however, is equally true; and when death has ensued, no amount of treatment will induce their retention, but, like other foreign substances and dead tissue, it will be disposed of by the processes by which nature accomplishes such work. A crownless root I consider far more apt to become diseased and die than one not deprived of that very essential portion, because, with the crown, it has lost that essential stimulant to good health, viz: exercise, and consequent irritation, inducing better circulation of the blood about it, and therefore better nutrition.

There are to-day few intelligent and conscientious practitioners in our profession who would extract an ulcerated superior incisor with crown complete, half, or even a larger portion, gone; but they would, by proper treatment, heal the same, fill the root and restore the crown to its normal shape with gold, sometimes even to disfigurement. I am sorry to believe, however, that many of these same practitioners would extract, did there not exist the opportunity to display an advertisement in the shape of a contour filling. It cannot be that the half crown restored with gold to its normal form in any manner assists in maintaining the healthy condition of the root, otherwise than as has been mentioned, by giving the necessary exercise, and thereby better circulation and nutrition.

If a root can be made healthy and capable of supporting a natural, dead crown, and fifty per cent. of this foreign material, why may it not support an artificial crown equally as well? I say dead crown, because I understand it to be admitted that the enamel and dentine of a devitalized tooth are dead, and that the tooth is only retained through the life of the cementum, in which the vitality is of so low an order that it tolerates the dead dentine within. This is certainly a very fortunate condition of things for those who advocate contour fillings in devitalized teeth, or the setting of pivot teeth upon such roots.

The first step to be considered is, of course, the preparation of the root for the reception of the artificial crown. If it be in a diseased condition, it must first be made healthy. My practice is, first, after removing a sufficient quantity of the remaining crown, to give free access, to enlarge the pulp canal, and, if there be no indications of active disease, use such remedies as will thoroughly disinfect the same; then pack with cotton loaded with the saturated solution of iodine in creasote, and seal tight. If no soreness arises after two or three days, I then feel safe in filling that portion of the canal above the hole which is to receive the pivot, either with gold, cotton dipped in liquid gutta percha, or Hill's stopping. I prefer the latter because of its indestructibility, and because it can be easily and thoroughly introduced, and if trouble arise it may be easily removed for further treatment. If an ulcer exist, disinfect as in the first case, and leave the root open or filled lightly with cotton for a day or two; then inject the solution of iodine and creasote through the foramen and fistula, if there be one. Unless it be a very obstinate case, one dressing of this character will suffice, leaving for a few days with a simple light cotton stopping, and as soon as the fistulous opening is healed, proceed, as before mentioned, to fill permanently.

The preparation of that portion of the root which is to receive the pivot may depend entirely upon the condition of the root and the kind of crown to be attached, and possibly upon the length of the patient's purse, for "the poor ye have always with you," and they cannot be turned away. It may be carefully and thoroughly filled, covering every portion of dentine, around a gold tube, which is to contain the pivot; or it may be simply drilled sufficiently large for the reception of the pivot, be it of either wood or metal. I have, when badly decayed, prepared as for a gold filling, and filled with amalgam, using for the tube platina instead of gold, because convinced that there is less galvanic action between the alloy and this metal, than exists between the alloy and gold. About three years ago I inserted two central incisors for a patient in this manner, which are at the present writing every way satisfactory to myself as well as to the patient.

The use of Wood's fusible metal as a root filling is dangerous as well as otherwise unsatisfactory—dangerous because the heat necessary to be applied is often sufficient to cause death to surrounding tissues, and consequent eventual loss of root—os-artificial and Guilois' cement are not sufficiently reliable as to their permanency, being in a large majority of mouths dissolved by the oral secretions. If the first method be decided upon, viz: the tubing and filling of the root, I proceed first to the manufacture of the tubing or hollow wire; selecting a piece of iron, or, what is better, steel wire the same size as the gold wire to be used for pivot, around which bend a piece of eighteen carat gold plate, fitting as closley as is possible with mallet or hammer; then reduce to the proper size and thickness by drawing the whole through a wire draw-plate. Having reached the desired size, remove the wire, and solder the joint to give stiffness in future use; then, with a screw-plate, cut a thread about as far as will be necessary for the case in hand—from one-half to three-eighths of an inch; then drill the root just large enough to admit the tube being firmly screwed into it, without using sufficient force to twist it. It is unnecessary to tap out this hole, as the thread upon the tube makes sufficient thread for itself, and holds firmer than when the tap is used.

These preparatory steps do not necessitate the filing away of the crown to the margin of the gums. In fact, it is the best not to do so until the rubber dam is adjusted, which may be carried sufficiently high to be out of the way, and secured by two small gold screws as nearly upon the mesial and distal surfaces as it is convenient to drill, tying above them a well waxed cord of silk; this will in nearly all cases retain the dam securely. This accomplished, that portion of the crown necessary to be removed may be filed away, or, what is better, cut away with proper shaped burs with any of the burring machines in use. Then, if the root be a sound one, cut a cone-shaped cavity in its end, with undercuts deep enough, which, with the thread upon the tube, will retain the filling firmly. If decayed, of course remove all disorganized tooth substance, as in any other case when preparing for a filling. Now screw the tube into place, leaving within it a piece of the wire over which it is made, to prevent injury to its inner surface, and proceed to fill. The filling should in all cases cover every portion of the root's end, and also be built down sufficiently far to leave, when filed off, a cap or plate of the necessary thickness for a solid foundation, and stiffness enough to remain in place. If the crown be already broken, or decayed away to the margin of the gum, a ferrule of gold or platina may be fitted around the end of the root, secured with gutta percha or *os artificial*, and the dam put on over this; or in comparatively dry mouths, if the parts be wiped dry and painted with the liquid gutta percha or collodion, thus effectually preventing the secretions from these parts giving trouble; using napkins, as in general filling, to control the flow of saliva.

These details will apply to the preparation of all roots where tubing is resorted to. If the filling be of amalgam, the rubber dam may be dispensed with. Complete dryness is, however, quite as essential for a perfect amalgam as for gold fillings.

The root prepared by any of the above or other methods, we next turn our attention to the crown to be attached and kind of pivot to be used.

Prof. Austen, after canvassing all the known materials used for pivots, concludes that wood is superior to all others, for the following reasons: First, that it holds the crown firmer; and secondly, because it does not, like metal, wear away the root. That when first introduced it does retain the crown very firmly, is true—no more so, however, than metal may be made to do. The second conclusion in its favor, or objection to metal, is not of sufficient importance to be considered. Any material firmly held in place will not wear the roots, or tube, sufficiently to be observed in at least one generation, and in these cases we do not work for anything beyond our own day and age. When by chance the holes in root and crown to be set are opposite, and the pivot straight, and the patient does not object to the odor always arising from a tooth set in this manner, wood answers a very good purpose for a limited number of years.

We all understand that constant dryness is one of the essentials in preventing decay of tooth substance. A wood pivot necessarily gives us constant moisture; in fact, it depends upon this property of absorbing moisture, in a great measure, for its firmness. Saturated with pure water even, it would hasten decay; but when saturated with such fluids as it must be in the mouth, what must be the result? Therefore, when permanence is to be one of the considerations, we must discard wood, and select a material less destructible. For reasons embodied above, in my own practice I have adopted the metal pivot, and will feebly endeavor to point out some of the ways in which it may be used.

If it be desirable to use the crown known as the pivot tooth, either a platina or stiff gold wire may be inserted into it by dipping the end of the wire into pulverized borax, inserting into the hole and flowing solder about it. No trouble will be experienced in accomplishing this, if it be remembered to keep the highest heat upon the tooth. The solder will fill the space about the wire, fitting into all the irregularities of the hole and holding very firmly. When the holes in root and crown are opposite, like the pivot of wood, this answers a very good purpose; and even when an obtuse angle is necessary in the pivot, it can be much easier fitted and be made stronger than the wood pivot. The attaching of a pivot of metal to the plain tooth requires more labor, but in my opinion, its superiority in all respects more than compensates for this. It is easily adjusted to conform to the other teeth, be the root before or behind the arch, whether the hole, owing to the direction of the root, be straight or at an angle, as well as possessing greater strength.

It is usual, first, to secure an impression of the end of the root and adjoining teeth, to make metal dies, and swedge a piece of plate to cover the end of the root; this to be punctured at the point covering the hole prepared for the pivot, and, when fitted, soldered to the pivot. Upon this plate the tooth is to be set, the two pieces cemented together with shellac and adjusted to the mouth, removed and soldered. If to be so made as to be removed at the will of the patient, the pivot must be made of two halves of half round spring wire, or split with a fine saw before inserting, the two parts being sufficiently separated to form a spring, which will retain the tooth in place. If to be securely fixed, the hole should be a little larger than the pivot, and the pivot should be coated with Hill's stopping or gutta percha, the root wiped dry, the pivot and coating warmed and forced home, and so held until the whole becomes cold; then trim away the surplus materials. This not only holds the pivot firmly, but fills the space between the plate and root, thus protecting the latter. Gutta percha I think preferable because it adheres closer to the root and pivot. During the past three years I have set many teeth in this manner: Preparing the root as for a wood pivot, take an impression, and upon the plaster cast burnish a plate of pure gold or platina, fix the pivot, and try it in the root. If the plate does not fit accurately, it may be fitted either with a burnisher or by mallet force, with a large pointed plugger; then attaching the tooth as above related, and the whole secured in its place with gutta percha.

I have seen roots upon which teeth set in this manner, after several years wearing, showed no signs of decay. This method saves time, and, therefore, money to the patient, when such an end is desirable, and gives a very substantial and satisfactory result.

Another very good suggestion upon this subject is given in the March No. of the *Cosmos*, 1873, which I cannot describe better than in the writer's own words:

" Instead of adapting a crown with a fixed pin of either wood or metal to a root, insert a metal pin in the root, make a filling of gold on the end of the root, and adapt the crown to that. There will only be exposed what appears to be a small filling along the margin of the gum. Our method is to insert an eighteen carat pin into the root, by means of threads cut on that part of the wire to be inserted, having the other end cleft with a fine saw, and protruding sufficiently to occupy the hole in the artificial crown. The wire having been firmly screwed into the root, and a proper crown selected and adjusted, enter two or three of Dr. Mack's small retaining screws, cut pieces from the heaviest gold, say No. 480, a little larger than the end of the root, with holes punched to correspond with the screws inserted in the root, lay on one after another, condensing with smooth faced instruments, occasionally paying special attention to condensing small pieces arouud the pins, maintaining the convexity of the root; then set the crown, and file and burnish down the protruding gold about the edges of the roots."

With this method, I do not understand it to be the intention of the writer to file the root below the margin of the gum. The capping of the end of the roots is made easy by the help of the rubber dam. This is, so far as the preparation of the root, somewhat similar to Dr. Mack's method; but for a crown he provides a patent tooth, with a slot for the reception of two or more screws, secured in the root, the slot to be filled with Wood's fusible metal, Guilois' cement, or other plastic material. This method fails, owing to the destructible nature of the materials used in uniting the crown to the root.

I have with me a couple of these patent teeth, that any who desire may examine. In the November No. 1872, *Cosmos*, there appears an article headed "Artistic Dentistry," by Dr. H. M. Webb, of Lancaster, Penn., in which he gives a description of what he terms grafting, which is, in fact, the restoration of the crown with gold, but faced with porcelain. It involves the same principles as the plans above given, viz: the protection and prevention of further decay of the root, and though by no means entirely new, is truly artistic and enduring. Such work was recommended several years since, by Dr. Dwinell, and I know of at least one bicuspid root thus provided with a crown some four or five years since, by a practitioner in Chicago, in the mouth of one now present. Although operations such as these are tedious to operator and to patient, as well as necessarily expensive, they possess advantages, and one is that a tooth when fractured obliquely and devitalized, it may be desirable to save that portion of the crown remaining, and as objections are urged against the exposure of so much gold, the porcelain face may be attached, firmly secured and backed up with cohesive gold, showing only a fine line of gold at the point of union. As the treatment of diseased teeth by extraction and replanting is at the present time being somewhat agitated, and has in many cases proved successful, I will relate two cases of extraction of roots with pivoted crowns, and then returned to the sockets, with results.

Dr. Meredith, of Cincinnati, publishes his experiences something as follows:

A patient called, desiring a pivot tooth set upon a root much diseased, and very loose, advised extraction, which was not consented to. A pivot tooth only would satisfy the patient. After much persuasion, and some compunctions of conscience, the doctor consented, proceeding to business, and succeeded to his satisfaction in the selection and setting of the substitute, except finding it somewhat too long, and in the attempt to remove it, for the purpose of shortening, extracted root and all. Fearing a brisk breeze if the patient discovered the mishap, he placed over the other teeth, and place where root had been, a napkin, requesting that it be retained there until his return, retired to the laboratory, shortened the tooth, cleaned the root properly, and returning, forced the root again into the socket, dismissing the patient. After a reasonable amount of soreness, this root became healthy, and does service at present.

Dr. Brophy, a practitioner of Chicago, related to me a case in practice, involving the same principles. He, however, extracted purposely a root, upon which an artificial crown had been some time worn, treated as is recommended for ulcerated teeth, and replanted. This also became a healthy organ, and bids fair to do good service for a number of years.

These cases I mention simply to show that treatment which is admissible for diseased roots bearing natural crowns may successfully be resorted to with roots bearing artificial crowns, whenever it would seem necessary or desirable to attempt to save them. The cases when such practice would seem to be necessary may be few and far between, still when they do present themselves, the experience of the gentlemen above mentioned may be of great value.

